# Supplementary material for: Preparation and Size Control of Efficient and Safe Nanopesticides by Anodic Aluminum Oxide Templates-Assisted Method
Source: Int J Mol Sci. 2021 Aug 3;22(15):8348. doi: 10.3390/ijms22158348 (PMC8347391; doi:10.3390/ijms22158348)
Supplement: Supplementary file 1 [file ijms-22-08348-s001.zip › Supplementary Materials.pdf]

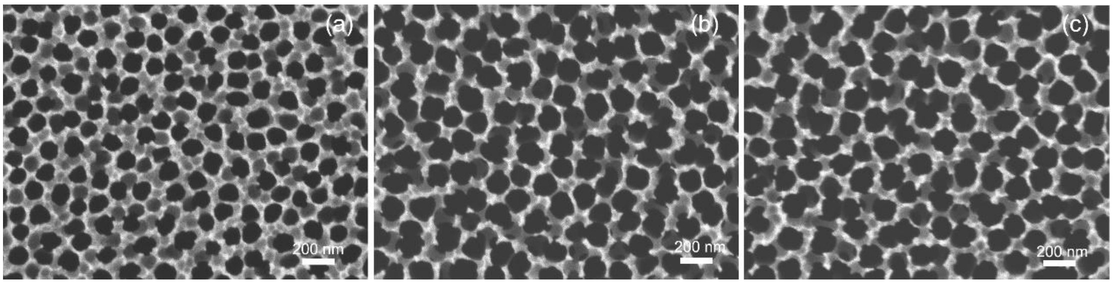

Figure S1. The SEM images of AAO templates after immersing in methanol, tetrahydrofuran and acetone.

(a) SEM image of AAO templates in methanol, (b) SEM image of AAO templates in tetrahydrofuran, (c) SEM image of AAO templates in acetone.

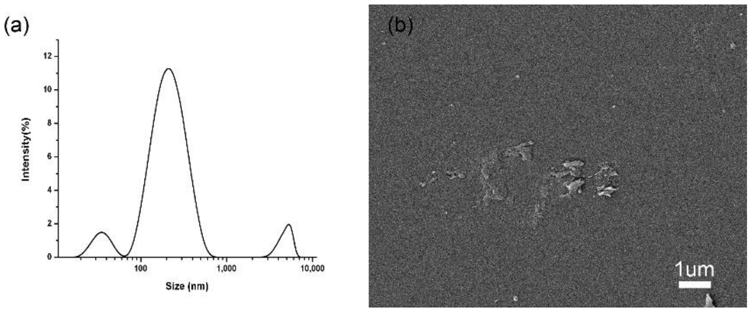

Figure S2. The DLS and SEM image of the buprofezin particles (BPs) using reprecipitation

(a) DLS image of BPs, (b) SEM image of BPs

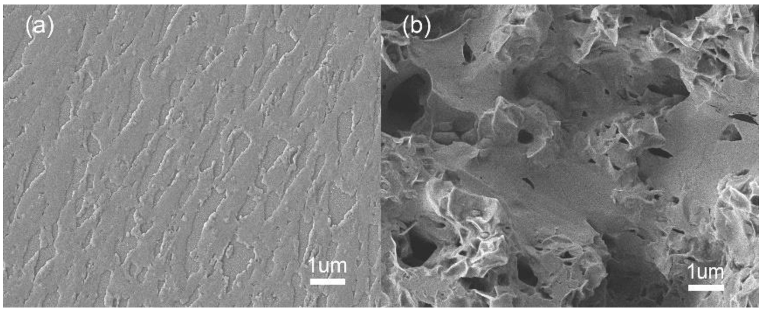

Figure S3. The SEM image of the AAO templates without drugs under alkaline condition and acidic condition

(a) SEM image of AAO templates in 0.1 mol/L NaOH solution (b) SEM image of AAO templates in 1 mol/L hydrochloric acid solution

Table S1. The comparison between AAO template method and reprecipitation methods

Characterization	AAO template method	Reprecipitation method
Particle size	Controllable	Uncontrollable
Uniformity	Monodispersity	Polydispersity
Dispersibility	Uniform dispersion	Uneven dispersion
Applicability for pesticide	High	General
