# Supplementary figures and images for: Preparation and Size Control of Efficient and Safe Nanopesticides by Anodic Aluminum Oxide Templates-Assisted Method
Source: Int J Mol Sci. 2021 Aug 3;22(15):8348. doi: 10.3390/ijms22158348 (PMC8347391; doi:10.3390/ijms22158348)

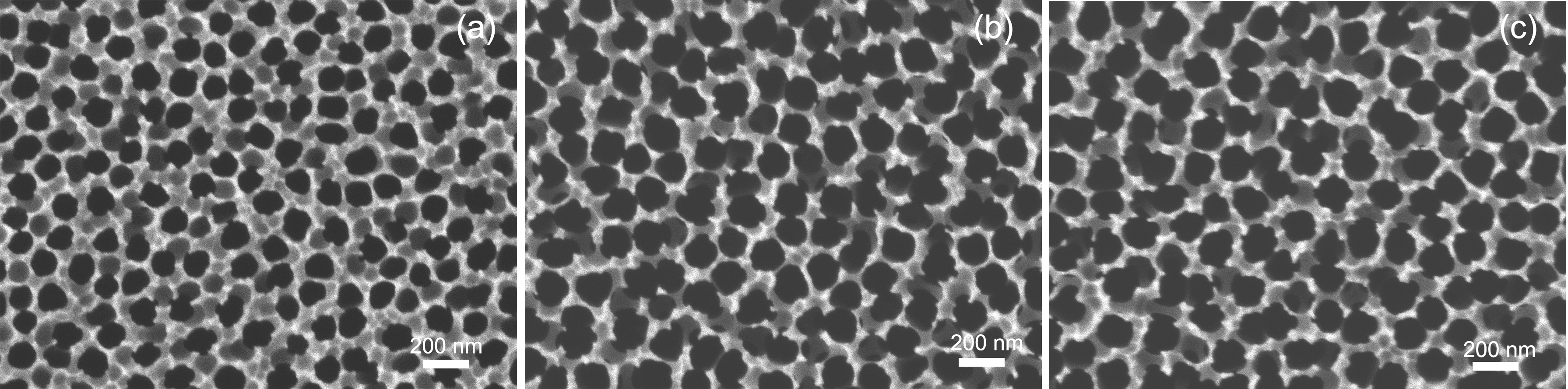

Supplement: Supplementary file 1 [file ijms-22-08348-s001.zip › Figure S1.tif]

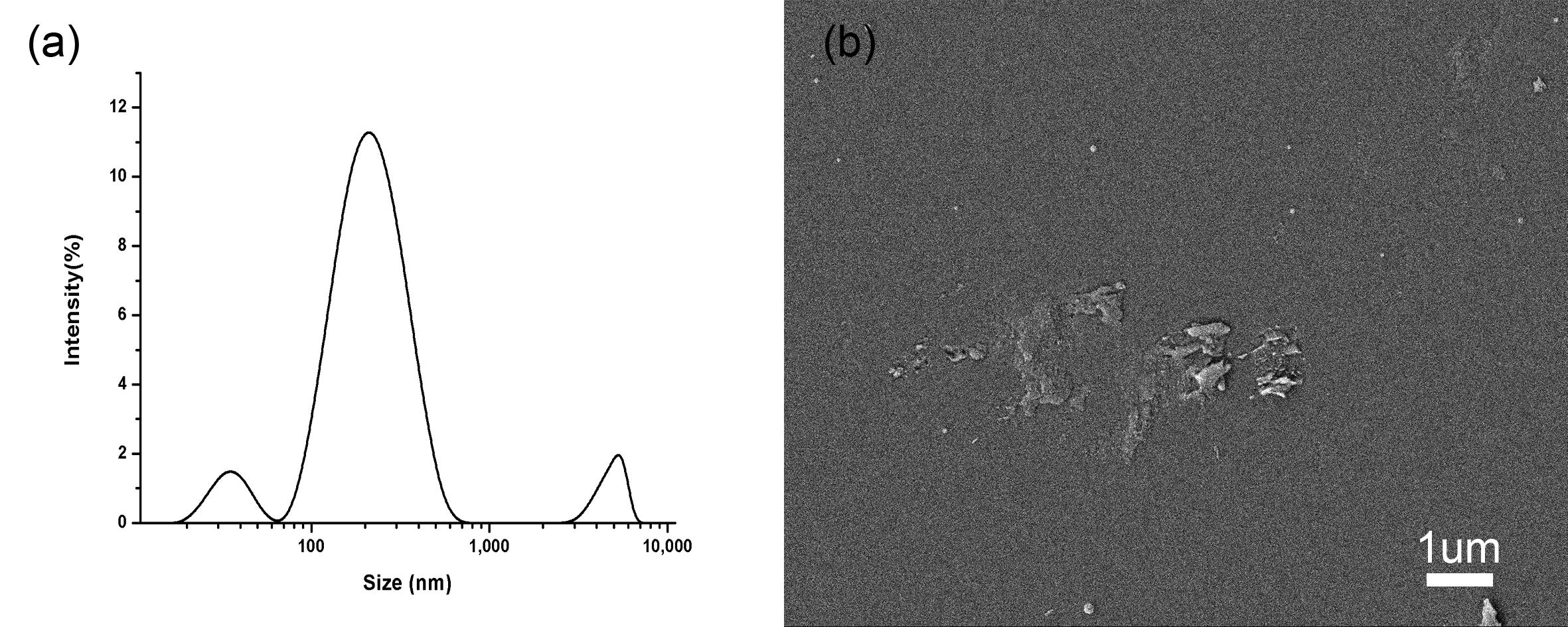

Supplement: Supplementary file 1 [file ijms-22-08348-s001.zip › Figure S2.tif]

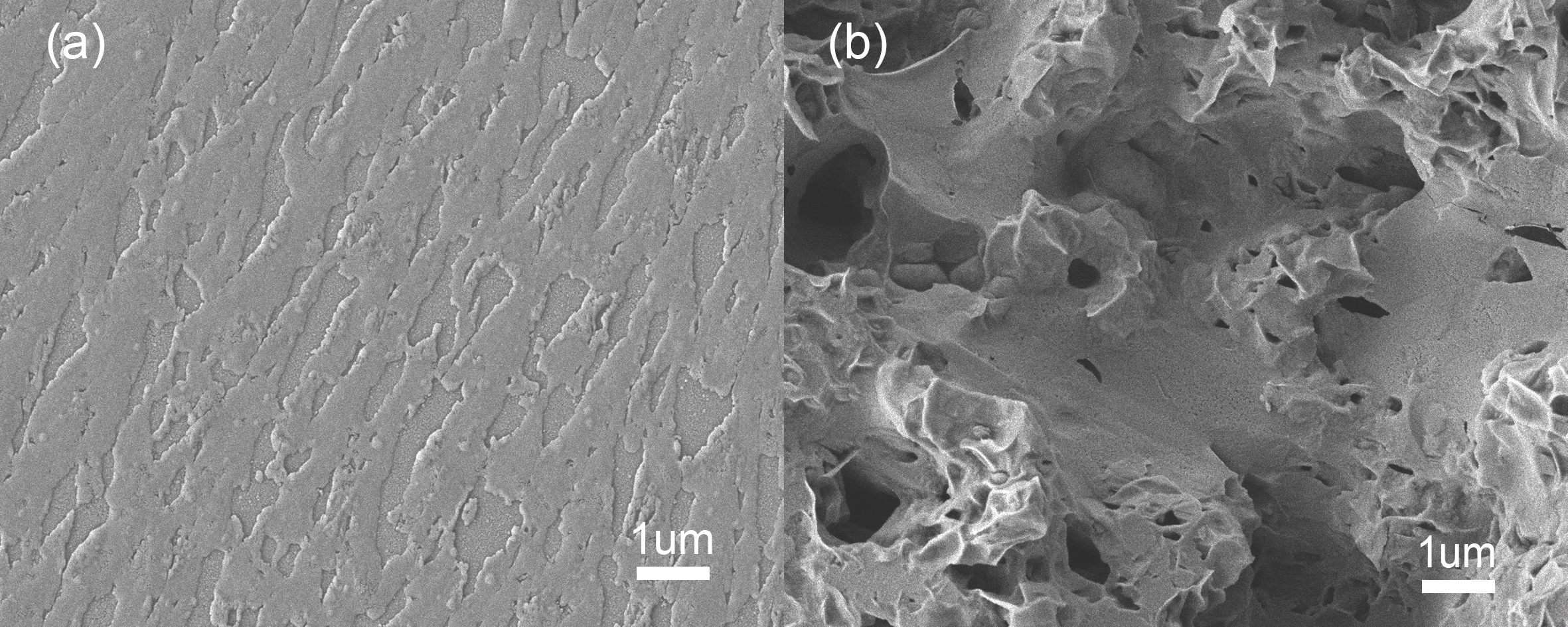

Supplement: Supplementary file 1 [file ijms-22-08348-s001.zip › Figure S3.tif]
